# A phase-plane analysis of localized frictional waves

**DOI:** 10.1098/rspa.2016.0606

**Published:** 2017-07-05

**Authors:** T. Putelat, J. H. P. Dawes, A. R. Champneys

**Affiliations:** 1 Department of Engineering Mathematics, University of Bristol, Bristol BS8 1UB, UK; 2 Department of Mathematical Sciences, University of Bath, Bath BA2 7AY, UK

**Keywords:** friction, rate-and-state, self-healing slip pulse, detachment front, global bifurcation

## Abstract

Sliding frictional interfaces at a range of length scales are observed to generate travelling waves; these are considered relevant, for example, to both earthquake ground surface movements and the performance of mechanical brakes and dampers. We propose an explanation of the origins of these waves through the study of an idealized mechanical model: a thin elastic plate subject to uniform shear stress held in frictional contact with a rigid flat surface. We construct a nonlinear wave equation for the deformation of the plate, and couple it to a spinodal rate-and-state friction law which leads to a mathematically well-posed problem that is capable of capturing many effects not accessible in a Coulomb friction model. Our model sustains a rich variety of solutions, including periodic stick–slip wave trains, isolated slip and stick pulses, and detachment and attachment fronts. Analytical and numerical bifurcation analysis is used to show how these states are organized in a two-parameter state diagram. We discuss briefly the possible physical interpretation of each of these states, and remark also that our spinodal friction law, though more complicated than other classical rate-and-state laws, is required in order to capture the full richness of wave types.

## 1. Introduction

Inhomogeneous frictional sliding between solid bodies is ubiquitous and characterized by multiple spatio-temporal scales, compelling and practical examples being earthquake mechanics [1] or brake squeal [2]. In a sense, friction may be seen as an additional fundamental force, an irreversible process by which kinetic energy is transferred from macroscales to the microscale and dissipated in the form of heat. In mechanical systems, it has been estimated that up to 1.4% of GDP could be saved by a better management of interfacial wear and frictional energy loses [3]. Not only a nuisance, friction is also beneficial in many situations, such as in mechanical dampers, brakes and clutches, and there are many works aimed at controlling friction from robotics to turbine blades [4–6].

Within engineering dynamics, it is common to use approximate friction laws such as those of Coulomb or Stribeck [7], which allow simple distinction between stick and slip, or more elaborate versions such as the LuGre model [8]. The validity domain of such simple models is at best questionable [9], and theoretical studies tend to be limited to either point contact or to where surfaces in contact are assumed to behave homogeneously, although see [10–15] for notable exceptions. Nevertheless, such approximations have proved useful in situations where friction is regarded merely as a loss mechanism on an otherwise macroscopic motion. Using such an approach, quite sophisticated mathematical theories of non-smooth mechanics have developed [16,17], which can predict complex temporal behaviours at the macroscale (e.g. [18,19]).

Tribology, on the other hand, being the detailed study of the wear, friction, adhesion and lubrication processes in the vicinity of interacting surfaces in relative motion, is a much more subtle topic that requires understanding of thermo-chemo-mechanical processes that occur at the microscale, or even below. In general, temperature dependence, micro-fluidics, molecular structure and the precise arrangement of surface asperities can all play a role in understanding the mechanisms that occur inside the contact region (e.g. [20–24]).

At a much larger length scale, theories of earthquake formation often rely on models of frictional contact between tectonic plates, see [1,25–27] for reviews. One of the things these models try to understand is how travelling *slip pulses* can be generated after years of inactivity or creep [14,28–31]. Moreover, the intricate nature and diversity of earthquake types which have been recorded over the past decade, including aseismic events, episodic tremors, slow and fast earthquakes [32,33], which together suggest a continuum of sliding modes, remain a challenge in terms of mathematical modelling and physical understanding. Finally, the question of how locked zones along creeping faults are created, the failure of which leads to classical earthquakes, is also very intriguing [34].

For example, Avouac and collaborators [35] made observations in the 2003 Chengkung earthquake that (i) ‘seismic slip occurred on a fault patch that had remained partially locked in the interseismic period, (ii) the seismic rupture propagated partially into a zone of shallow aseismic interseismic creep but failed to reach the surface, and (iii) the aseismic afterslip occurred around the area that ruptured seismically’. A possible interpretation of these observations could be in terms of a new frictional slip mode that we describe in the present paper as a *stick pulse*. A stick pulse corresponds to a narrow sticking zone travelling against a slipping background and which might be identifiable as the seed of an earthquake.

Another major issue in the wider field of friction modelling is that experimental characterization of dynamic interfacial friction forces is fraught with difficulty. Apart from the branch of nanotribology, most results are restricted to macroscale measurements, which are typically characterized by scatter of data and lack of repeatability. One approach used to gain a quantitative match with theory, is to use high-resolution imaging or computation of the *asperity map* of the surface, to understand its precise roughness characteristics [36–38]. The predictive power of this approach, limited by the computing power available, is somehow questionable because asperities continually change with wear, temperature and loading conditions. A notable exception is the work of Fineberg and co-workers (e.g. [39–41]), who use ingeneous optical techniques to measure the detailed patterns of slip and stick between two transparent plates being slowly sheared against each other via a constant tangential force. The results suggest the existence of both fast and slow fronts and slip pulses. Another interesting study is that of Behrendt *et al.* [42] which, although not experimental, uses high-resolution numerical simulations to study a two-dimensional elastic block in contact with a rigid surface using a local Stribeck-like friction law. Different small-scale stick, slip and lift-off waves are found to occur as the macroscale slipping velocity varies between 0.5 and 40 mm s^−1^.

The ethos of the present paper is to attempt to bridge the gap between the macroscopic non-smooth dynamical systems approach of friction modelling and the detailed tribological point of view. We are also motivated by phenomena that might be universal across time and force scales, from earthquakes interseismic periods of decades to squeal oscillations in the kilohertz range. To that end we study a canonical, dimensionless, problem at the next order of complexity from point or homogeneous regional contacting behaviour, namely the existence of travelling fronts and pulses of stick-like and slip-like behaviour between two homogeneous frictional surfaces.

In contrast to most studies, the main purpose of our article then is to consider a general theory allowing at once for the generation of propagating slip- and stick-pulse, together with propagating slip and stick fronts, in models for regional interfacial contact. We emphasize that this study shows that these localized slip patterns can exist over a continuum range of wavespeeds; the range of wavespeeds can be explicitly computed as a function of the driving stress. We propose that this theory sheds new light on the processes responsible for the large variability of earthquake duration and frequency spectrum [32,33].

Our analysis builds on previous work [43,44] where the full three-dimensional elastodynamic problem is reduced to that of the idealized situation of a long thin elastic plate sliding against a rigid substrate. In [43,44], detachment front solutions were computed and shown to result from the non-monotonic crossover of the steady-state friction characteristic from velocity-weakening to velocity-strengthening regimes (e.g. [45–49]). Such failure modes were interpreted as slow slip events and argued to explain incipient sliding friction along spatially extended contact as experimentally monitored [39,40]. Going much further, using the classical travelling-wave reduction of partial differential equations (PDEs) posed on an unbounded domain, we shall give precise descriptions for these localized states in terms of different types of connecting orbits [50].

We finally note that a variety of solitary waves in the form of slip pulses or fronts were also numerically found within the one-dimensional continuum limit of the Burridge–Knoppoff model [51], either with velocity-dependent non-smooth friction laws [52–56] or the Ruina rate-and-state law [31].

This paper is organized as follows. Section 2 introduces and justifies our choice of friction law, namely a non-monotonic rate-and-state law that we analysed in a number of different mechanical settings in previous work [48,57,58]. Such models are well-motivated experimentally, yet are analytically tractable and avoid the non-smoothness associated with strictly Coulomb-like models. Section 3 introduces the geometry and mechanics of the problem to be studied. It is shown how the equations of motion reduce through long-wave approximation to a one-dimensional nonlinear wave equation, and through non-dimensionlization we identify its key parameters. We perform linear stability analysis and show numerically that the system supports travelling waves. Such waves are described by a two-dimensional dynamical system having a slow–fast multiple time-scale structure that is analysed in the phase plane, through a combination of analytical and numerical bifurcation analysis. Section 4 presents a complete exploration of the existence regions of various kinds of response, including wavetrains, stick and slip pulses and fronts between regions of homogeneous stick or slip. Our results are summarized in terms of two-parameter diagrams involving dimensionless parameters that represent the speed of travelling waves, and the applied shear stress. Finally, §5 comments on the physical applicability of the work, shows how our extended rate-and-state model has the minimal complexity necessary to capture the phenomena at hand, and suggests avenues for future work.

## 2. Rate-and-state friction model

At the heart of this paper lies the use of a recently proposed model for rate-and-state friction that captured many experimental observed effects that are not captured by non-smooth Coulomb-like models. We give here only a brief motivation, and refer the reader to recent work by the first two authors, e.g. [58,59] and references therein, for further details and discussion.

Basic Coulomb-like models classify material contacts via a single constant, the *static coefficient of friction* *μ*_s_ which is the maximum ratio of tangential interfacial shear stress *τ* to normal stress *σ* that can sustain *stick*. Alternatively, the bodies are said to *slip*, with the ratio equal to some other *kinetic coefficient of friction* *μ*_k_ which may, in general, be a (non-monotonic) function of relative velocity *v* in general (in a so-called Stribeck’s Law [7]). However, engineers have long recognized the limitations of such an approximation. In the 1950s, Rabinowicz, Bristow and co-workers, see e.g. [60–62] argued from experimental evidence that most (*μ*,*v*) curves have a finite positive slope for low *v*, with the stick state better being described as a slow *creep* and what is called *μ*_s_ being some quantity near the maximum of the curve. Rabinowicz [62,63] also highlighted dependence on past sliding history and introduced the idea of a *critical slip distance* before any effect of the previous slip rate history has faded away. Equivalently, these memory effects determine a *persistence length* over which friction preserves its static value before dropping to its kinetic value for impulsively accelerating frictional interfaces. In the absence of such memory effects, the empirical assumption of a time-dependent static friction coefficient and a velocity-dependent kinetic friction coefficient has been shown to fail to reproduce the basic physics of stick–slip oscillations [62].

In a seminal 1966 paper, Brace & Byerlee [64] later on proposed that, rather than by brittle fracture, earthquakes could arise from recurrent stick–slip instabilities along pre-existing fault planes [65]. This shift of paradigm has caused the study of rock friction to flourish, propelled in particular by the work of Dieterich [66] and Ruina [67,68], who introduced the so-called rate-and-state formulation for a frictional interface. In such a formulation, memory effects are encoded in an internal variable *ϕ*(*t*) that characterizes the state of the contact region and quantifies its resistance to slip, originally thought of as representing average lifetime of a population of interfacial contacts [66,69] even though different microphysical origins could actually be conjectured [59]. In the rate-and-state framework of friction, the time evolution of the interfacial state *ϕ*(*t*) is determined by an empirical evolution equation whose precise mathematical expression remains an open question. We note, however, that the use of a first-order kinetics for the interfacial state evolution is mathematically justified whenever *ϕ* is interpretable as an *n*th statistical moment of the friction force [70]. On pure mathematical grounds, it has also been argued that piecewise-smooth descriptions of friction such as Coulomb’s need to be smoothed out by introducing an evolution equation for some hidden variable in order to account for any departure from steady sliding and hysteretic effects [18]. Originally, ad hoc choices of state evolution laws, such as (5.1), were motivated by fitting the frictional stress relaxation observed in response to sudden changes in driving velocity within ‘slide-hold-slide’ experiments [66,68]. The physical and experimental foundations of rate-and-state models can found in references [69,71–73].

Rate-and-state models of friction are thus phenomenological in essence and assume that the interfacial shear stress *τ* is determined by nonlinear equations of the form

2.1 $$\tau =F(v,\phi ,\sigma )\;\text{and}\;\frac{d\phi }{dt}=-\frac{G(v,\phi ,\sigma )}{t_{*}},$$

where the interfacial slip rate *v*=d(*δu*)/d*t* corresponds to the time derivative of the slip jump *δu*:=[[*u*]] across the frictional interface, t_*_ is the characteristic time scale over which the interfacial state relaxes to equilibrium, and *σ* represents the normal stress applied to the interface. Different models correspond to different choices for the functions *F* and *G*, models with several internal variables, say *ϕ*_*i*_, being possible, at least conceptually [68].

Most realizations of (2.1) assume that *F* is simply proportional to the normal stress, as in Coulomb friction, so that *F*=*μ*(*v*,*ϕ*)*σ* with

2.2 $$\mu =\mu_{*}+a\;\ln\left(\frac{v}{V_{*}}\right)+b\;\ln\left(\frac{\phi }{\phi_{*}}\right),$$

where V_*_ and ϕ_*_:=*L*/V_*_=t_*_ are reference values of the slip rate and the interfacial state so that the value of the kinetic friction coefficient *μ* takes the value *μ*=μ_*_ in steady sliding at *v*=V_*_. The dimensionless material parameters *a* and *b*, which are of the order of 10^−2^ for most materials, can be fitted to experimental measurements (e.g. [71,74]) and represent the amplitude of the frictional response to sudden velocity variations [75]. A characteristic *memory length* *L*, which is connected to Rabinowitz’s persistence length [58], is usually introduced in place of the time scale t_*_ in connection with the definition of (2.1)_2_, as we show below. Note meanwhile that the phenomenological analytical form (2.2) is well justified both experimentally and theoretically from physical first principles, see [59] and references therein.

In the absence of any *ϕ* dynamics, e.g. in steady sliding for which *v*:=*V* , the interfacial state must reach an equilibrium *ϕ*_ss_ so that *G*(*V*,*ϕ*_ss_,*σ*)=0, which in combination with (2.2), yields the steady-state friction curve *μ*_ss_(*V*):=*μ*[*V*,*ϕ*_ss_(*V*)].

In particular, ‘regularized generalizations’ of the ageing law, defined by (2.2) with (5.1), can be built with expressions of the form

2.3 $$\begin{array}{rl} & \frac{F}{\sigma }=\mu (v,\phi )=a\;\sinh^{-1}\;\left[\epsilon^{-1}\left(\frac{v}{V_{*}}\right){\left(c+\phi \right)}^{b/a}\right],\;\epsilon :=2\;\exp\;\left(-\frac{\mu_{*}}{a}\right)\ll 1\\ \;\text{and}\; & G(v,\phi )=\frac{\sinh[\phi -\phi_{\mathrm{ss}}(v)]}{t_{\phi }(v)}, \end{array}\}$$

where *c* is an additional material constant describing the residual strength of the interface at high slip velocities, which requires fitting. *t*_*ϕ*_(*v*) and *ϕ*_ss_(*v*) are (dimensionless) functions of the sliding velocity which describe the interfacial time scale and steady interfacial state. As in our previous work [58], we propose a ‘spinodal’ version of the friction law, taking

2.4 $$t_{\phi }(v)=\frac{R}{1+R}\phi_{\mathrm{ss}}(v)\;\mathrm{and}\;\phi_{\mathrm{ss}}(v)=\frac{1+R}{1+Rv/V_{*}},$$

where *R*≫1 is the ratio of the much slower time scale t_**_ to t_*_; t_**_ is the characteristic time scale for relaxation of the interfacial state variable in stationary contact, allowing for interfacial state saturation [76]. With (2.4), the steady-state friction curve *μ*_ss_(*V*) is non-monotonic and has a local maximum and a minimum located at

2.5 $$\frac{v_{\max}}{V_{*}}\approx \frac{a}{R(b-a)}\;\mathrm{and}\;\frac{v_{\min}}{V_{*}}\approx \frac{b/a-1}{c}.$$

We remark that models (2.3) and (2.4) are regularized both mathematically in the sense that the logarithmic singularity of (2.2) at v→0 is avoided, and physically in the sense that unbounded weakening at moderate sliding speed (i.e. greater than or equal to 1 mm s^−1^) is prevented. In addition, if the $\sinh^{-1}$ expression for *μ* can be justified from microphysical theories of friction featuring thermally activated Eyring rate processes (see [59] and references therein), the sinh expression for *G* is somehow arbitrary, however, physically justified in ensuring a strong interfacial healing that prevents unphysical and unbounded acceleration of the interface in quasi-stationary contact (see [58] for more details).

Note that, compared to simple Coulomb-like models, there are many rate-and-state models and they often contain many ad hoc parameters. See, for example, the recent studies [74,77,78] that discuss how results from a pin-on-disc experiment allow the determination of parameters and discrimination between different rate-and-state models. Indeed, in [74] it was shown that the extra complexity associated with some rate-and-state models was necessary in order to replicate the qualitative nature of the frequency response curves.

In §5a, we return to the question of choice of friction law and we show how simpler monotonic or unregularized models do not capture the rich diversity of solution types that have been observed experimentally, and which are produced with the above friction law defined by (2.3) and (2.4). Alternative non-monotonic rate-and-state models can be found in [43,44,48].

## 3. A model problem for a thin sliding slab

### (a) Formulation

**Figure 1.**
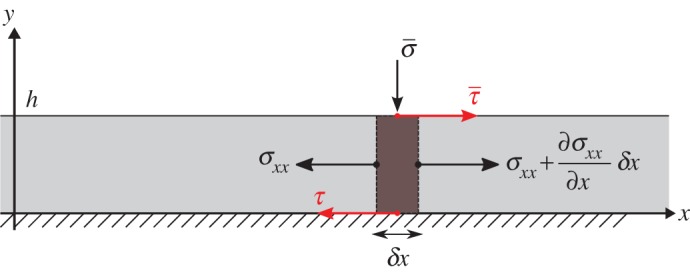
Definition sketch of the elastic plate and forces balance on a cross-section of infinitesimal width *δx*. (Online version in colour.)

Consider an infinite elastic plate of thickness *h*, density *ρ*, Young’s modulus *E*, shear modulus S and Poisson’s ratio *ν*, that is subject to a spatially uniform constant normal stress $\bar{\sigma }$ and is driven from the top by a constant shear stress $\bar{\tau }:=\bar{\mu }\bar{\sigma }$. The bottom of the plate is assumed to slide undeformed but with friction on a flat and horizontal rigid foundation (figure 1). Provided the wavelength of the elastic longitudinal wave propagating in the plate is large compared with its thickness, Lamb [79] showed that the distribution of the longitudinal stress and displacement components *σ*_*xx*_ and *u* is uniform across the plate’s cross-section. Following [80], the plate equation of motion can then be derived from a consideration of force balance in a cross-section of infinitesimal width *δx* and of unit length in the transverse direction (figure 1), yielding

3.1 $$(\rho h\delta x)u_{,tt}=\left(\sigma_{xx}+\sigma_{xx,x}\right)h-\sigma_{xx}h+(\bar{\tau }-\tau )\delta x.$$

Furthermore, assuming uniformity of the vertical component of the stress across the plate, i.e. $\sigma_{yy}\equiv \bar{\sigma }$, Hooke’s Law implies that the vertical strain component $v_{,y}=(-\lambda u_{,x}+\bar{\sigma })/(\lambda +2S)$. Hence the longitudinal stress *σ*_*xx*_=(λ+2S)*u*_,*x*_+λ*v*_,*y*_ reads

3.2 $$\sigma_{xx}=\frac{4S(\lambda +2S)u_{,x}+\lambda \bar{\sigma }}{\lambda +2S}.$$

The first and second Lamé coefficients are denoted λ and S (i.e. the shear modulus). Combining (3.2) with (3.1), the dimensional equation of motion of the plate becomes

3.3 $$u_{,tt}=c_{l}^{2}u_{,xx}+\frac{\bar{\tau }-\tau }{\rho h},$$

where the longitudinal wave speed *c*_*l*_ is defined by $c_{l}^{2}=\bar{E}/\rho$ where $\bar{E}:=E/(1-\nu^{2})$.

There are, in fact, alternative ways of deriving the slab equation (3.3). For example, we can use a ‘shallow-layer’ approximation to the full three-dimensional elasto-dynamic equations in which the slab displacement corresponds to the vertical averaging $u(x,t):=\int_{0}^{h}\tilde{u}(x,y,t)\;dy$ [43]. Alternatively, an asymptotic thin plate theory has been developed which shows that (3.3) arises at leading order. The details will appear elsewhere.

The model (3.3) is completed by using a friction law of the form (2.1) to express *τ* in terms of the sliding velocity *u*_,*t*_ and the state variable *ϕ*. By using the scales *h*/*c*_l_, *h*, V_*_h/*c*_l_ and $\bar{E}(V_{*}/c_{l})$ for non-dimensionalizing time, space, displacement and stress, respectively, the rate-and-state nonlinear dynamics of the plate is then governed by the dimensionless system

3.4 $$\zeta (u_{,tt}-u_{,xx})+\mu (u_{,t},\phi )=\bar{\mu }\;\mathrm{and}\;\phi_{,t}=-rG(u_{,t},\phi ),$$

where we use the regularized spinodal form so that *F* and *G* are given by (2.3) and (2.4). The two dimensionless parameters appearing in (3.4) are

3.5 $$\zeta =\frac{\rho c_{l}}{\bar{\sigma }/V_{*}}\;\text{and}\;r=\frac{h/c_{l}}{t_{*}}.$$

The parameter *ζ* compares the wave impedance to the friction impedance, which estimates the order of magnitude (for *μ*≈1) of the kinetic friction force that resists sliding at speed V_*_. Alternatively, *ζ* can be thought of as the reciprocal of dimensionless normal pressure measured in units of the stress scale $\bar{E}V_{*}/c_{l}$. The parameter *r* compares the characteristic time scale of information propagation carried by the slab elastic waves, over a distance of order *h*, to the characteristic rejuvenation time scale of the interfacial state (of order 1 for most materials). In practice, we expect both parameters to be small: *ζ*,*r*≪1.

### (b) Uniform sliding states and their stability

Spatially uniform and steady sliding states (*u*_,*t*_(*x*,*t*),*ϕ*(*x*,*t*))=(*v*_0_,*ϕ*_0_) of the plate correspond to solutions of the nonlinear system

3.6 $$\mu (v_{0},\phi_{0})=\bar{\mu }\;\mathrm{and}\;G(v_{0},\phi_{0})=0,$$

and are fully determined by the value of the dimensionless driving stress $\bar{\mu }$, independently of *ζ* and *r*. Using (2.3) we can rewrite (3.6) as

3.7 $$\mu_{\mathrm{ss}}(v_{0})\equiv \mu (v_{0},\phi_{\mathrm{ss}}(v_{0}))=\bar{\mu }\;\mathrm{and}\;\phi_{0}=\phi_{\mathrm{ss}}(v_{0}).$$

The number of solutions to (3.7) depends on the shape of the steady-state friction curve *μ*_ss_(*v*). Unlike the case of a monotonic friction law, which allows for a unique uniform steady-state sliding, the case of the spinodal law (2.3) and (2.4) allows for three possible uniform steady sliding solutions whenever $\mu_{M}\leq \bar{\mu }\leq \mu_{m}$. We interpret these three steady states as representing ‘stick’, ‘creep’ and ‘slip’, in order of increasing steady-state velocity. Typical orders of magnitude for these three states are 10 nm s^−1^, 1 μm s^−1^ and 10 mm s^−1^, referring to the steady-state velocity curve sketched in figure 10.

It is straightforward to show that only the stick and slip states, which lie on the velocity-strengthening branches of the friction curve, are stable to spatially homogeneous perturbations. In general, to examine the possibility of wave-like instabilities we consider the behaviour of infinitesimal perturbations to a uniform state, writing

$$(u_{,t},\phi )=(v_{0},\phi_{0})+(\hat{v},\hat{\phi })\exp(pt+ikx),$$

where $k\in R^{+}$ is a spatial wavenumber and *p*=*s*−i*ω* gives the corresponding growth rate. The dispersion relation is as usual given by the determinant of the linearization of (3.4):

$$\left(\begin{array}{cc} p^{2}+k^{2}+\frac{p\mu_{,v}}{\zeta } & \frac{\mu_{,\phi }}{\zeta }\\ rpG_{,v} & p+rG_{,\phi } \end{array}\right),$$

where, similar to before, *μ*_,*v*_ denotes the partial derivative of the function *μ* with respect to *u*_,*t*_ evaluated at (*v*_0_,*ϕ*_0_). The dispersion relation takes the form

3.8 $$p^{3}+\left(rG_{,\phi }+\frac{\mu_{,v}}{\zeta }\right)\;p^{2}+\left(k^{2}+\frac{rG_{,\phi }\mu_{\mathrm{ss}}^{\prime }}{\zeta }\right)\;p+rG_{,\phi }k^{2}=0,$$

where the slope of the friction curve *μ*_ss_′:=d*μ*_ss_(*v*_0_)/d*v*_0_ appears by the chain rule.

In the spatially homogeneous case *k*=0, we obtain the quadratic equation *ζp*^2^+(*rζG*_,*ϕ*_+*μ*_,*v*_)*p*+*rG*_,*ϕ*_*μ*_ss_′=0, where *r*,*ζ*≪1, which can be rewritten more clearly as

3.9 $$r^{2}\zeta^{2}\;{\left(\frac{p}{r}\right)}^{2}+(r\zeta G_{,\phi }+\mu_{,v})r\zeta \;\left(\frac{p}{r}\right)+r\zeta G_{,\phi }\mu_{\mathrm{ss}}^{\prime }=0$$

so that the leading root can be estimated to be *p*/*r*=−*G*_,*ϕ*_*μ*_ss_′/*μ*_,*v*_+*O*(*rζ*).

Neutral stability curves can be obtained by investigating the two cases *p*=0 and *p* complex but with a zero real part. In the first case, we observe that *p*=0 occurs only when *k*=0, so that neutrally stable modes having *k*≠0 are always oscillatory. Therefore, we look for modes for which *p*=−i*ω*, $\omega \in R^{+}$. The real and imaginary parts of the cubic (3.8) then lead to

3.10 $$\omega^{2}=\left(\frac{r\zeta }{r\zeta +\mu_{,v}/G_{,\phi }}\right)\;k^{2}=k^{2}+\left(\frac{r}{\zeta }\right)\;G_{,\phi }\mu_{\mathrm{ss}}^{\prime }.$$

Combining these two expressions for *ω*^2^ yields the wavenumber *k*_*c*_ and frequency *ω*_*c*_ for neutrally stable modes:

3.11 $$k_{c}^{2}=\omega_{c}^{2}\;\left(1+\frac{\mu_{,v}}{r\zeta G_{,\phi }}\right),\;\text{where}\;\omega_{c}^{2}=-\frac{r^{2}G_{,\phi }^{2}\mu_{\mathrm{ss}}^{\prime }}{\mu_{,v}}.$$

Applying the Routh–Hurwitz criterion, we conclude that steady-state sliding is stable (i.e. ℜ(*p*)<0 for all *k*) to all perturbations with wavenumbers *k* for which $k^{2}>k_{c}^{2}$. In summary, uniform states on the velocity-strengthening branches of *μ*_ss_(*v*) are stable to all wavenumbers, whereas uniform states on the velocity-weakening branch are unstable to sufficiently long-wavelength perturbations for which *k*<*k*_*c*_. As Ruina [67] remarks, ‘the growth of instabilities is, then, insensitive to small wavelength (stiff) perturbations and very sensitive to long wavelength (soft) perturbations’ (see also [81,82]).

**Figure 2.**
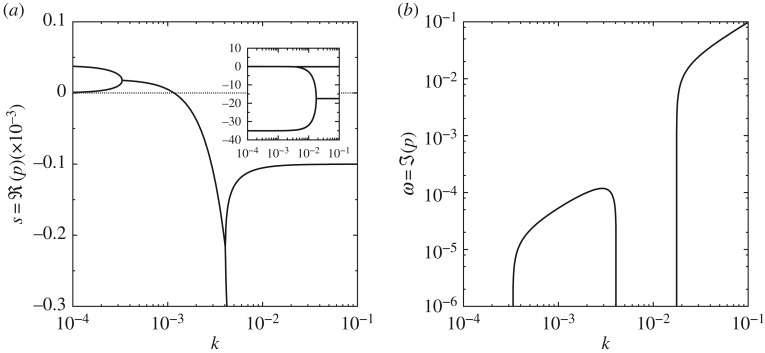
Dispersion curves for the spinodal law (2.3): (*a*) growth rates of perturbations with wavenumber *k*; (*b*) temporal frequencies of perturbations with wavenumber *k*. Parameters: *v*_0_=10, *r*=10^−5^, *ζ*=0.1.

**Figure 3.**
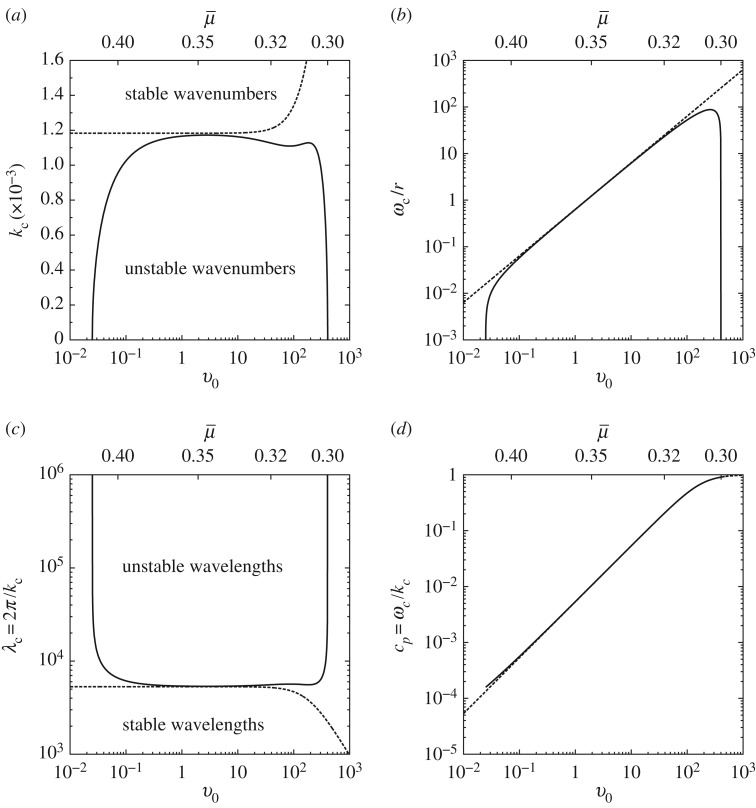
(*a*) Stability region of perturbation wavenumbers *k* as a function of slip rate *v*_0_ as defined by (3.11). (*b*) Neutral angular frequency *ω*_*c*_ as defined in (3.11). (*c*) The corresponding neutral wavelength λ_*c*_:=2*π*/*k*_*c*_. (*d*) The neutral phase velocity *c*_*p*_:=*ω*_*c*_/*k*_*c*_: note this will correspond to the Hopf bifurcation locus H in figure 6. The dashed lines indicate the corresponding curves obtained from the Dieterich–Ruina monotonic laws (2.2) with (5.1). Parameters: *r*=10^−5^, *ζ*=0.1.

Figures 2 and 3 illustrate these results using the material parameter values listed in table 1 and the following dimensionless parameter values:

3.12 $$r=10^{-5}\;\mathrm{and}\;\zeta =0.1,$$

which correspond to material that has a thickness of the order of a few millimetre that is subjected to a normal pressure of the order of 10 Pa.

**Table 1.** Material parameter values used in the friction laws (2.3) and (2.4). These parameter values fit the experimental data obtained for the frictional properties of Bristol paper board [46] and paper sheet [83].

μ_*_	*a*	*b*	*L* (μm)	V_*_ (m s^−1^)	*c*	*R*	*ρ* (kg m^−3^)	*E* (GPa)	*ν*
0.369	0.0349	0.0489	0.9	10^−6^	10^−3^	100	550	4	0.3

The linear stability analysis above is useful also to estimate the validity of the long wavelength approximation of the wave equation (3.3). It is clear from figure 3 that the critical wavenumber for the monotonic Dieterich Law (cf. §5a), $k_{c}^{2}=(r/\zeta )(b-a)(1+r\zeta v^{2}/a)$, provides a good upper bound for the instability domain. Hence the long-wave approximation, λ_*c*_≫1, remains valid whenever *r*/*ζ*≪4*π*^2^/(*b*−*a*), i.e. a confining pressure $\bar{\sigma }\ll [4\pi^{2}/(b-a)](L/h)\bar{E}∼1\;\mathrm{GPa}$, which is the case for both engineering systems and geophysical contexts. Equivalently, the thickness of the layer must satisfy $h\ll [4\pi^{2}/(b-a)](\bar{E}/\bar{\sigma })L∼10^{6}/\bar{\sigma }$. For typical values of the confining pressures $\bar{\sigma }$ ranging from a few GPa up to of the order of MPa, the latter expression suggests that the thin slab approximation remains valid for thicknesses ranging from a few millimetre up to of the order of metre.

### (c) Multiple time-scale analysis of travelling waves

In addition to uniform steady states, the differential equations (3.4) are expected to support travelling waves. In this section, we investigate the existence of travelling waves and their generation in Hopf bifurcations from the uniform steady-state solutions. Without loss of generality, we assume that waves travel to the left, so that we introduce a travelling wave co-ordinate *z*=*r*(*t*+*x*/*V*) with travelling wave velocity *V* >0. System (3.4) then reduces to a planar system of nonlinear ordinary differential equations for the unknown functions *v*(*z*)=*ru*_,*t*_ and *ϕ*(*z*):

3.13 $$\gamma \dot{v}=\mu (v,\phi )-\bar{\mu }\;\mathrm{and}\;\dot{\phi }=-G(v,\phi ),$$

where a dot now denotes d/d*z*. We find that the parameters *V* , *ζ* and *r* combine naturally into a single dimensionless parameter *γ*:

3.14 $$\gamma :=\frac{r\zeta (1-V^{2})}{V^{2}},$$

which is a monotonically decreasing function of the travelling wave speed *V* . Note that the regime *γ*≥0 corresponds the interval 0≤*V* ≤1 of subsonic wave speeds (note the longitudinal wave speed *c*_l_ is scaled to unity in the non-dimensionalization leading to (3.4), which means that *V* is measured in units of *c*_l_).

System (3.13) has a natural slow–fast asymptotic structure because *r*,*ζ*≪1 and hence *γ*≪1, provided *V* is considered to be fixed and non-zero. Alternatively, we can consider the limit *γ*≪1 to be equivalent to the limit V→1, that is the limit of fast travelling waves. This naturally defines *z* as a slow ‘time scale’ so that *v* evolves quickly until *μ*(*v*,*ϕ*) is close to $\bar{\mu }$ while *ϕ* evolves slowly. In the limit γ→0 the slow variable *ϕ* is governed by the slow subsystem or *reduced problem*

3.15 $$0=\mu (v,\phi )-\bar{\mu }\;\mathrm{and}\;\dot{\phi }=-G(v,\phi ).$$

For small *γ*, the full system (3.13) evolves slowly provided it stays within a small neighbourhood of the *critical manifold* defined by

3.16 $$\mu (v,\phi )=\bar{\mu },$$

which couples the evolution of *v* to that of the state variable *ϕ*: inverting (3.16) defines the relation $v=v_{⋆}(\phi ;\bar{\mu })$.

By contrast, if we rescale the equations on the fast time scale $\hat{z}=z/\gamma$ we obtain the fast system

3.17 $$v^{\prime }=\mu (v,\phi )-\bar{\mu },\;\phi^{\prime }=\gamma G(v,\phi ).$$

The natural approximation of this fast system is then to consider that the evolution of the fast variable *v*, when *ϕ* is constant, is given by the fast subsystem or *layer problem*

3.18 $$v^{\prime }=\mu (v,\phi )-\bar{\mu },\;\phi^{\prime }=0.$$

The one-dimensional dynamics along the critical manifold of the slow subsystem (3.15), governed by

**Figure 4.**
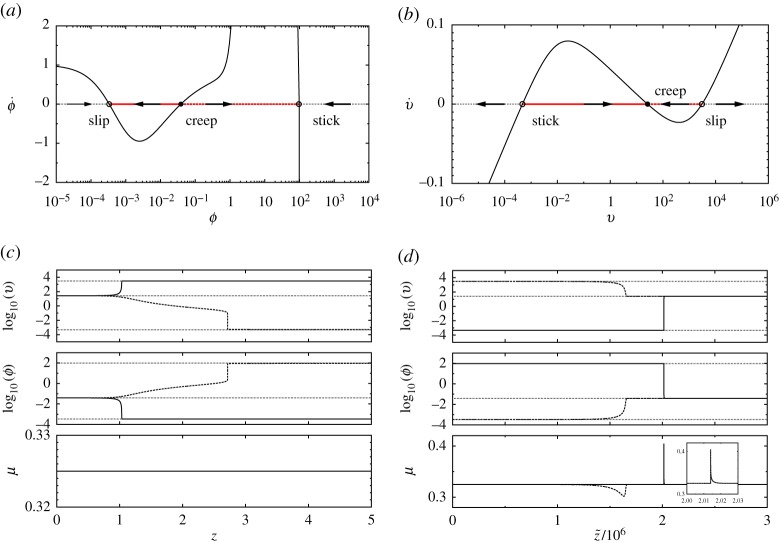
The one-dimensional qualitative dynamics of the slow subsystems (3.15) and (3.22)_2_ for the fast fronts (*a*–*c*) and slow fronts (*b*–*d*) (moving to the left). The solid and dashed lines depict the fixed point connection type corresponding to ‘detachment’ and ‘attachment’ front solutions, respectively. These fronts belong to the regions v and viii in figure 7. Parameter: $\bar{\mu }=0.325$. The symbol open circles denotes the stick and slip equilibria and closed circles the creep equilibrium. (Online version in colour.)

3.19 $$\dot{\phi }=-G[v_{⋆}(\phi ;\bar{\mu }),\phi ]=:-g(\phi ;\bar{\mu }),$$

is of importance as it provides a first insight into the solution types of the full system (3.13). For a spinodal friction model such as ours, $g(\phi ;\bar{\mu })$ is of course a non-monotonic function of *ϕ* whenever $\mu_{M}\leq \bar{\mu }\leq \mu_{m}$ and three equilibria describing homogeneous sliding exist. As shown in figure 4*a*, the changes of sign of $\dot{\phi }$ indicate that the creep equilibrium point is unstable, whereas the stick and slip equilibria are stable. In terms of travelling wave solutions, this means that travelling fronts connecting the creep equilibrium to either the stick or slip equilibria can exist over open intervals in both the driving stress $\mu_{M}\leq \bar{\mu }\leq \mu_{m}$, and the wave speed *V* , whose set of values cannot be determined at this level of analysis. The connection between the creep and slip (respectively, stick) states represents a sharp jump between the corresponding interfacial slip rates, which can be interpreted as a generic *detachment front* (respectively, *attachment front*), see figure 4*c*. From this computation, a rough estimate of the front width is *δx*∼*δzV*/*r*≫1, whereas *δz*=*O*(1) and *r*≪1, which agrees with long wavelength approximation of this slab model. Physically, the dynamics of these fronts, which can travel *fast* with *V* =*O*(1), is determined by the interfacial state evolution law in such a way that the interfacial slip rate is slaved to the interfacial state so that the friction stress remains uniform and equal to that of the driving.

An alternative distinguished limit exists for the case of very slow travelling waves, where $V\ll \sqrt{r\zeta }$, so that instead of being small, *γ*≫1. We define *ϵ*:=*γ*^−1^≪1, and then define a new slow time variable $\tilde{z}$ with $z=\tilde{z}/\epsilon$, so that the fast and slow systems, respectively, are now as follows:

3.20 $$\left\{ \begin{array}{l} \dot{v}=\epsilon [\mu (v,\phi )-\bar{\mu }]\\ \dot{\phi }=-G(v,\phi ) \end{array}\right.\;\text{and}\;\left\{ \begin{array}{l} v^{\prime }=\mu (v,\phi )-\bar{\mu }\\ \epsilon \phi^{\prime }=-G(v,\phi ), \end{array}\right.$$

where now *v* represents the slow variable, *ϕ* the fast variable and the critical manifold being defined by

3.21 $$\phi =\phi_{\mathrm{ss}}(v).$$

The fast and slow systems (3.20) are analogous to the slow and fast systems (3.13) and (3.17), and the corresponding layer and reduced problems then read

3.22 $$\left\{ \begin{array}{l} \dot{v}=0\\ \dot{\phi }=-G(v,\phi ) \end{array}\right.\;\text{and}\;\left\{ \begin{array}{l} v^{\prime }=\mu (v,\phi )-\bar{\mu }=\mu_{\mathrm{ss}}(v)-\bar{\mu }\\ 0=-G(v,\phi ) \end{array}\right.$$

Similarly, the slow dynamics along the critical manifold (3.21) governed by (3.22)_2_ allows for the existence of generic *slow* detachment and attachment fronts, but now, connecting either the stick or slip states to the creep state (figure 4*b*–*d*). These slow fronts travel with $V=o(\sqrt{r\zeta })$ and hence are characterized by an interfacial state which has time to relax to its steady-state value, becoming in turn enslaved to the evolution of the interfacial slip rate controlled by inertia. In contrast to the fast fronts, these slow fronts are consequently associated with a pulse shape friction stress spatial distribution whose width is estimated to be $\delta x∼\delta \tilde{z}\sqrt{\zeta /r}\gg 1$.

We finally note that these different scalings of the problem indicate that dynamics is likely to be very ‘stiff’, with trajectories squeezed together in regions of phase space, and occasionally very rapid changes in the shape of typical orbits as parameters vary. Such phenomena could perhaps be fruitfully analytically tackled using Fenichel’s geometric singular perturbation theory [84]; we leave this to be the subject of future work. In the following sections, we present a detailed phase-plane and bifurcation analysis which shows that the full system (3.13) can exhibit a rich variety of solution types between the two asymptotic regimes of travelling fronts described here.

## 4. Detailed phase-plane analysis

We now study the bifurcation structure of travelling wave solutions to the system (3.13) using a combination of analytical and numerical methods. All numerical computations were carried out using the continuation software AUTO [85,86]. Except where otherwise stated, we use the spinodal law (2.3)–(2.4) with the material parameters as given in (3.12). We investigate the solution structure using the shear stress $\bar{\mu }$ and the travelling wave speed *V* as bifurcation parameters.

### (a) Overall bifurcation structure

**Figure 5.**
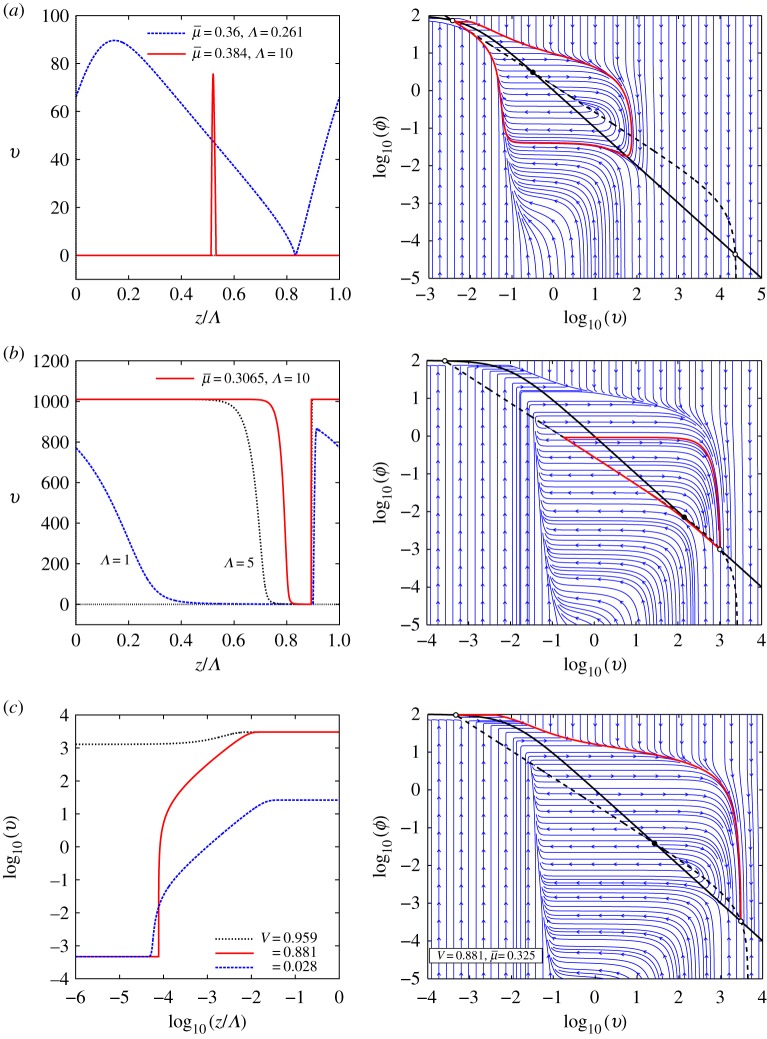
Examples of three different kinds of localized solution trajectory. (*a*) A slip pulse on a background of homogeneous stick (red curve containing a long section near zero), together with single periods of periodic slip waves of large amplitude and long but finite periods. (*b*) A stick pulse on a background of uniform slip (red curve containing a long section near a constant value of 1000) together with nearby long-period periodic travelling waves. (*c*) Three types of detachment fronts: (i) the solid red line indicates heteroclinic orbits between the low-velocity and high-velocity saddle points, (ii) the dotted black line corresponds to a fast generic front connecting the creep to the slip uniform states, (iii) the dashed blued line corresponds to a slow generic front connecting the stick to the creep uniform states. These generic fronts (ii) and (iii) correspond to the singular limit fast and slow fronts in figure 4*a*,*d* and *b*,*d*. In each case (*a*)–(*c*), the left-hand panel gives the profile of the localized wave, and of periodic solutions close to it, and the right panel shows the solution superimposed on the (*v*,*ϕ*) phase plane. See text for further details. Note that the scaling factor *Λ* in the left-hand panels corresponds to the period of the periodic orbits computed with AUTO. As *Λ* becomes large, the computed periodic orbits give a good numerical approximation of the localized solutions. In the phase portraits of the right-hand panel, the dashed and solid black lines, respectively, are the *v* and *ϕ* nullclines of system (3.13); they intersect at the three equilibria of (3.13) where the symbol open circle denotes the stick and slip saddle points and closed circle the creep equilibrium. The solid red lines depict numerical approximations of the saddle connections, pulse and front. (*a*) Slip pulse: *V* =0.1, $\bar{\mu }=0.384$, $\log_{10}(\gamma )=-4.00$. (*b*) Stick pulse: *V* =0.6, $\bar{\mu }=0.3065$, $\log_{10}(\gamma )=-5.75$. (*c*) Detachment fronts: *Λ*=0.3/*V* .

Figure 5 illustrates different kinds of spatially localized solution to the travelling wave system (3.13). The left-hand panels of each part of figure 5 show profiles of three different kinds of *localized* wave: (i) slip pulses, (ii) stick pulses, and (iii) detachment fronts. The interpretation and properties of these spatially localized solutions are described later. The right-hand panels in each part show how these waves appear as trajectories in the phase plane. In this and subsequent figures, the critical manifold (3.16) is indicated by a dashed line which coincides with the *v*-nullcline, whereas the *ϕ*-nullcline is indicated by a solid line. Equilibria (corresponding to uniform sliding states) are indicated by the solid black and white circles.

**Figure 6.**
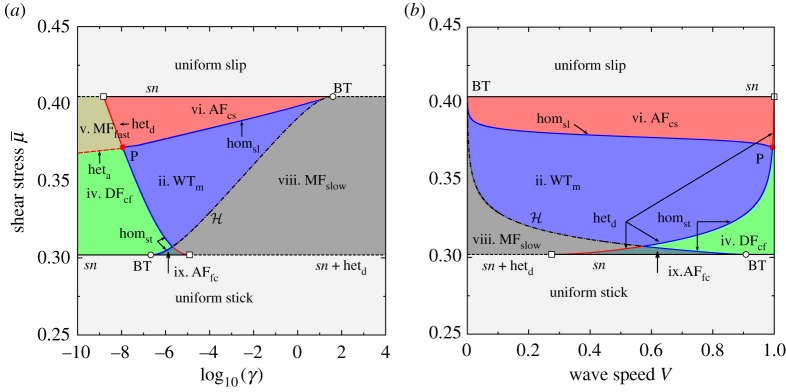
Fundamental phase diagram in the form of a two-parameter bifurcation diagram of the travelling wave system (3.13) under the spinodal rate-and-state friction model (2.4). As (*a*) *γ* versus applied shear stress $\bar{\mu }$ and (*b*) wave speed *V* against $\bar{\mu }$. Nomenclature: the global bifurcation lines of saddle–saddle connections are highlighted by the red lines (fronts) and blue lines (pulses): slip pulses (hom_sl_), stick pulses (hom_st_), detachment fronts (het_d_), attachment fronts (het_a_), see also figure 8. The local bifurcation lines are depicted with black lines: Hopf bifurcation locus (H), saddle-node bifurcation loci at the local extrema of friction (*sn*). Codimension two bifurcation points: Bogdanov–Takens (BT) bifurcation point, open circle), non-central saddle-node heteroclinic bifurcation point (open squares) [86]. Acronyms and numbered parameter regions correspond to those in figures 7 and 9: stick–slip wave-trains (WT_*m*_), generic detachment (DF) and attachment (AF) fronts. The attached subscripts refer to the type of generic connections, e.g. DF_cf_ for detachment fronts connecting the creep equilibrium (*c*) to the slip equilibrium (*f*).

**Figure 7.**
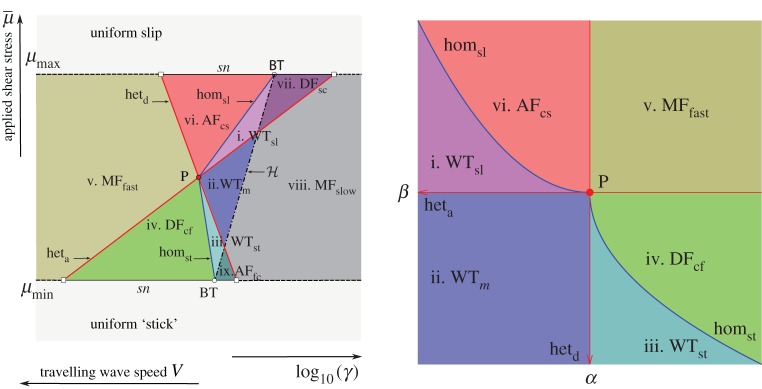
(*a*) Qualitative version of the bifurcation diagram in figure 6, unfolding the various bifurcation curves that appear to overlie each other due to the slow–fast nature of the true diagram. Note that the curves separating regions iii and iv, and regions i and vi are generically expected to lie tangent to other lines as shown in (*b*); they are shown as straight lines here for clarity. (*b*) Unfolding of the codimension-two point P as described in §4e. The phase portraits corresponding to the numbered regions are depicted in the following two figures 8 and 9.

**Figure 8.**
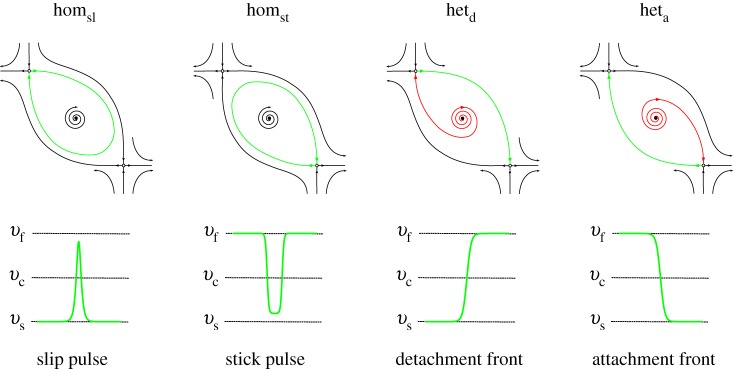
Main examples of qualitative phase portraits and waveforms corresponding to the saddle–saddle connections between the equilibrium points of (3.13). We denote their corresponding uniform slab slip rates by (*v*_s_,*v*_*c*_,*v*_*f*_), solutions of $\mu_{\mathrm{ss}}(v)\equiv \mu (v,\phi_{\mathrm{ss}}(v))=\bar{\mu }$, *G*(*v*,*ϕ*_ss_(*v*))=0.

**Figure 9.**
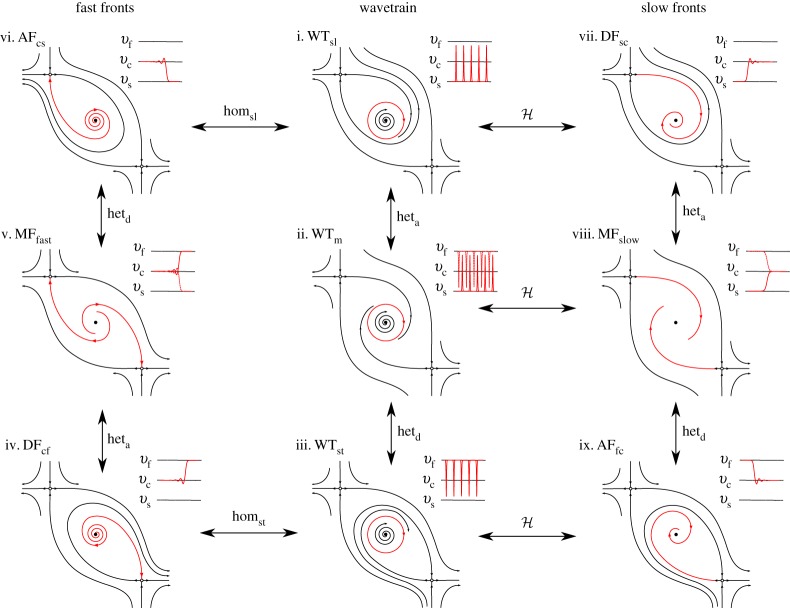
Qualitative phase portraits and sketch waveforms corresponding to the generic connections between the equilibrium points of (3.13). Acronyms are explained in figure 6.

Figure 6 depicts the two-parameter bifurcation diagram of the system, computed numerically using AUTO, showing curves of codimension-one bifurcations as lines in the $\left(\gamma ,\bar{\mu }\right)$ and $\left(V,\bar{\mu }\right)$ planes: these are equivalent representations due to relation (3.14). Figure 7*a* is a topologically equivalent version of the bifurcation diagrams shown in figure 6 that links the numerical bifurcation results to the theoretical analysis summarized in figure 7*b*. These two figures therefore are the core of our results and illustrate how the numerical and analytic investigations together enable us to provide a complete summary of the bifurcations associated with the existence of uniform states and travelling waves (figures 8 and 9).

Uniform states exist for all values of *γ*>0 and shear stress $\bar{\mu }$. For sufficiently high shear stress $\bar{\mu }>\mu_{\max}=\mu_{\mathrm{ss}}(v_{\max})\approx 0.4$ or sufficiently low shear stress $\bar{\mu }<\mu_{\min}=\mu_{\mathrm{ss}}(v_{\min})\approx 0.3$, there is a unique uniform state. Saddle-node bifurcations occur on the lines $\bar{\mu }=\mu_{\max}$ and $\bar{\mu }=\mu_{\min}$ associated with the local extrema of the friction characteristics.

**Figure 10.**
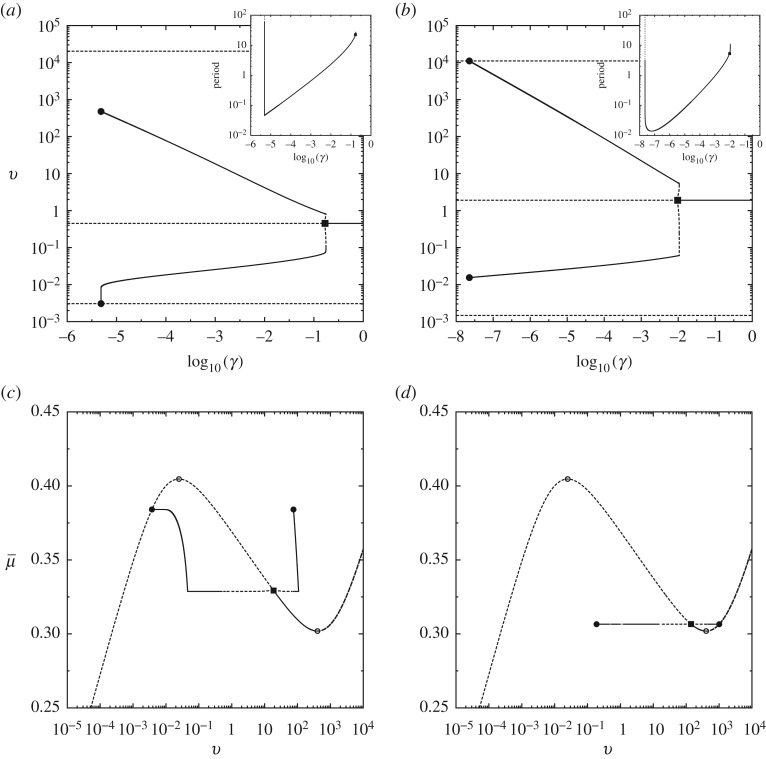
Numerical continuation of wavetrain solutions in *γ* for fixed $\bar{\mu }$ (*a*,*b*) and $\bar{\mu }$ for fixed *V* (*c*,*d*), with the maximum and minimum amplitude super-imposed on the *μ*_ss_ versus *v* curve. In *a*,*c* (respectively, *b*,*d*) column are typical bifurcation diagrams leading to the slip (respectively, stick) pulse homoclinic orbit. Here the Hopf bifurcation is illustrated via a solid square, folds by hollow circle and homoclinic orbits by a solid circle. Stable and unstable portions of the solution branch are illustrated by solid and dashed lines, respectively. Inserts show the divergence of the period as the homoclinic orbit is approached. (*a*) $\bar{\mu }=0.38$, (*b*) $\bar{\mu }=0.36$, (*c*) *V* =0.1 and (*d*) *V* =0.6.

For $\mu_{\min}<\bar{\mu }<\mu_{\max}$, there are therefore three distinct uniform states due to the spinodal nature of the friction model. The middle state on the velocity-weakening branch of the steady-state friction curve, i.e. the ‘creep’ sliding state, can become unstable along a Hopf bifurcation curve, which is precisely the neutral stability line that was computed in §3b (figure 3) as presented in §4b. The bifurcation produces a periodic travelling wave train that exists for values of *γ* below, or equivalently wave speeds *V* above, this curve (marked H in figures 6 and 7*a*). With increasing *V* or decreasing *γ*, the branch of periodic orbits is destroyed in one of two kinds of homoclinic bifurcation (blue lines in figures 6 and 7), leading to either localized slip or stick pulses that in phase space are homoclinic orbits to the slip or to the stick equilibrium points, respectively. See also the phase portraits in figure 5 and bifurcation diagrams in figure 10 for examples of such homoclinic orbits.

The Hopf bifurcation curve (H) terminates in Bogdanov–Takens (BT) bifurcation points where it meets one of the two saddle node lines, the location of which is indicated by a hollow circle in the figures. A standard unfolding of such a point (e.g. [87]) shows that the curves of homoclinic orbits must also originate from each of these points. In the interior of the spinodal region, the two homoclinic orbits meet at a codimension-two global bifurcation point (P), represented by a solid circle, where a complete heteroclinic cycle exists between the stick and the slip equilibria. An unfolding of that point is presented in §4e; although this kind of analysis is, in general, very well known, we could not find a direct reference in the literature to this specific case. In the following subsections, we comment in turn on each of the three main kinds of motion: wave trains of periodic travelling waves, pulses and fronts.

### (b) Periodic orbits: travelling waves

Spatially periodic wave trains arise from Hopf bifurcation points located on the velocity weakening branch of the steady-state friction curve. Study of the roots of the Jacobian matrix of (3.13) shows that the bifurcation point occurs at a critical value ${\bar{\mu }}_{h}$ of the shear stress $\bar{\mu }$ that provides the external driving, where ${\bar{\mu }}_{h}$ solves the equation

4.1 $$\mu_{,v}({\bar{\mu }}_{h})=\gamma G_{,\phi }({\bar{\mu }}_{h}),$$

which defines the locus (H) of the Hopf bifurcation points shown in figures 6 and 7). Note that (4.1) naturally follows from the neutral stability curve (3.11)_1_ derived in §3b after substituting the definition *V* :=*ω*_*c*_/*k*_*c*_ of the phase velocity of a linear wave. A good approximation to the critical stress is given by ${\bar{\mu }}_{h}\approx \mu_{*}+(a-b)\ln(v_{h})$, in which the critical slab slip rate $v_{h}=\sqrt{a/\gamma }$ follows from (4.1) using the classical Dieterich–Ruina Laws (5.1).

To go beyond, numerical continuation of the branch of periodic orbits indicates that there is typically one of two kinds of behaviour observed on varying $\bar{\mu }$ for different wave speeds *V* . These behaviours are illustrated in figure 10.

For sufficiently low wave speeds *V* , the behaviour of the bifurcating branch is qualitatively always as illustrated in figure 10*a*. The Hopf bifurcation gives birth to a branch of small-amplitude limit cycles that leads to a *canard explosion* (e.g. [88]) over an exponentially small range of the parameter $\bar{\mu }\approx {\bar{\mu }}_{h}$. The limit cycles are known as canard solutions of (3.13) and their amplitude and period increase significantly over this small range of parameter values as the limit cycle follows close to a segment of the slow manifold.

A typical canard cycle can be divided into three consecutive phases, as a consequence of the slow–fast asymptotic structure of (3.13). The fast phase defines the front of the velocity spike and corresponds to the sharp acceleration of the slab material points, whose dynamics is governed by (3.18) to leading order. Such an acceleration is accompanied by a decrease in the value of state variable (dynamic rejuvenation) since *ϕ*≫*ϕ*_ss_(*v*) until a maximum slip rate is reached. Subsequently, the slip rate decreases throughout an intermediate phase along the *ϕ*-nullcline during which the interfacial state is forced to remain close to its equilibrium value *ϕ*_ss_(*v*). As a result, the deceleration is associated with an increase of *ϕ* leading to the slow phase of the cycle along the critical manifold (the *v*-nullcline). The dominant contribution to the period of the limit cycle is from this final phase, *ϕ*, because *ϕ*≪*ϕ*_ss_(*v*). Eventually, the branch of relaxation oscillations terminates in a homoclinic bifurcation where the limit cycle collides with the low-velocity stick equilibrium point. For higher *V* , as in figure 10*b* the branch of limit cycles terminates in a homoclinic bifurcation in which it collides with the high-velocity slip equilibrium point, before any relaxation oscillation can occur.

### (c) Homoclinic orbits: travelling localized pulses

Homoclinic orbits, such as those that exist on the curves of homoclinic bifurcations shown in the $\left(\gamma ,\bar{\mu }\right)$ plane in figure 6, correspond to localized travelling pulses in the original (*x*,*t*) coordinates [89].

The orbit homoclinic to the equilibrium point with the lower value of *V* corresponds to a *slip pulse*: the interface creeps slowly, at the rate given by that of the ‘stick’ equilibrium, except in a short spatial region where the interface sees a velocity spike. The homoclinic orbit for moderate to large values of *V* corresponds to a *stick pulse*. Here, the whole interface slides at the rate associated with the high velocity saddle point while a localized patch of creeping material points is travelling along the interface.

### (d) Heteroclinic orbits: fronts

A different kind of localized travelling solution is a *front* solution that describes a transition in space between low- and high-speed regions of slip. In the phase plane for our ODE system, such a solution corresponds to a heteroclinic orbit, asymptotic to two different equilibrium points as *z* tends to ±∞ [89]. These particular solutions correspond to heteroclinic orbits which connect two different saddle points. For the model (2.3) and (2.4), there are two front solutions of particular interest.

The first of these correspond to a heteroclinic orbit formed by the intersection of the unstable manifold of the low velocity saddle point and the stable manifold of the high velocity saddle point. We interpret this ‘upper’ heteroclinic orbit as a *detachment front* since it describes a division of the slab into a part behind the front which is almost at rest while far ahead of the front the slab is moving rapidly (figure 5*c*).

The second (lower) heteroclinic orbit is the reverse: and intersection between the unstable manifold of the high velocity saddle point and the stable manifold of the low velocity saddle point. The rear of the slab is now sliding rapidly while ahead of the front the slab is moving rapidly. We interpret such a front as an *attachment front*.

We remark that numerically it is difficult to continue paths of some of the homoclinic and heteroclinic orbits, due to numerical stiffness caused by the slow–fast nature of the system. Nevertheless, we have confirmed the location of these curves as depicted in figure 6 through a series of one-parameter continuation runs of periodic orbits and noting points of divergence to large period.

### (e) The codimension-two point P

It is interesting to observe in figure 6 how all four bifurcation branches het_d_, het_a_, hom_sl_ and hom_st_ emerge from the organizing centre P, marked by a solid red dot, which represents a codimension-two heteroclinic cycle. We analyse the dynamics near P by constructing return maps describing trajectories in neighbourhoods of the two-saddle points involved in the heteroclinic cycle. This methodology and general notation is standard, going back to Poincaré and Shil’nikov; examples of this kind of calculation can be found in [90,91]. Interestingly, despite its simplicity we are unaware of a reference to this specific calculation in the literature.

Consider a planar system having two hyperbolic saddle points *p*_1_ and *p*_2_ with local coordinates aligned along eigenvector directions (*x*_1_,*y*_1_) and (*x*_2_,*y*_2_), respectively. Let the eigenvalues be −*c*_1_,*e*_1_ and *e*_2_,−*c*_2_, respectively, where *c*_1,2_,*e*_1,2_>0 denote the contracting and expanding eigenvalues at the saddle points. We further assume that at parameter values (*α*,*β*)=(0,0) there exists a heteroclinic cycle composed of two connecting orbits between the saddle points: one heteroclinic connecting orbit leaves *p*_1_ tangent to the *y*_1_>0 direction and approaches *p*_2_ tangent to the *y*_2_>0 direction. The other heteroclinic orbit leaves *p*_2_ tangent to the *x*_2_<0 direction and approaches *p*_1_ tangent to the *x*_1_>0 direction.

In terms of our travelling wave ODE system, the saddle point *p*_1_ corresponds to the low-velocity (stick) equilibrium, whereas *p*_2_ corresponds to the high velocity (slip) equilibrium point. The analysis proceeds by constructing leading-order approximations to the dynamics in two regimes: within small boxes |*x*_*j*_|,|*y*_*j*_|≤*d* near each saddle point, where the flow can be assumed to be linear (after a suitable coordinate transformation, due to hyperbolicity), and near each unstable manifold where we use the underlying differentiability of the vector field to make a Taylor series expansion. These two regimes lead to ‘local’ and ‘global’ maps between boundaries of these small boxes.

Accordingly, we define the sections (box sides) *Σ*_1_:={*x*_1_=*d*}, *Σ*_2_:={*y*_1_=*d*}, *Σ*_3_:={*y*_2_=*d*} and *Σ*_4_:={*x*_2_=−*d*}.

Consider first the local map $\Pi_{1}:\Sigma_{1}\to \Sigma_{2}$ near *p*_1_, where the flow is well approximated by the linear flow ${\dot{x}}_{1}=-c_{1}x_{1}$, ${\dot{y}}_{1}=e_{1}y_{1}$. Consider the trajectory starting from $\left(x_{1},y_{1})=(d,{\hat{y}}_{0}\right)$ and arriving at $\left(x_{1},y_{1})=({\hat{x}}_{1},d\right)$ at time *T*_1_>0. Integrating the ODEs we obtain *x*_1_(*t*)=*d* e^−*c*_1_*t*^, $y_{1}(t)={\hat{y}}_{0}\;e^{e_{1}t}$. At time *t*=*T*_1_, we can compute that

$$T_{1}=\frac{1}{e_{1}}\;\ln\;\left(\frac{d}{{\hat{y}}_{0}}\right)\;\text{and hence}\;{\hat{x}}_{1}=d\;{\left(\frac{d}{{\hat{y}}_{0}}\right)}^{-c_{1}/e_{1}}.$$

Similarly, consider the local map near *p*_2_ given by $\Pi_{3}:\Sigma_{3}\to \Sigma_{4}$ for which ${\dot{x}}_{2}=e_{2}x_{2}$, ${\dot{y}}_{2}=-c_{2}y_{2}$. We take the initial condition to be $\left(x_{2},y_{2})=({\hat{x}}_{2},d\right)$, so that $x_{2}(t)={\hat{x}}_{2}\;e^{e_{2}t}$, *y*_1_(*t*)=*d* e^−*c*_2_*t*^. At time *t*=*T*_2_, we define the trajectory to hit the point $\left(x_{2},y_{2})=(-d,{\hat{y}}_{2}\right)$ on *Σ*_4_, so that

$$T_{2}=\frac{1}{e_{2}}\;\ln\;\left(-\frac{d}{{\hat{x}}_{2}}\right)\;\text{and hence}\;{\hat{y}}_{2}=d\;{\left(-\frac{d}{{\hat{x}}_{2}}\right)}^{-c_{2}/e_{2}}.$$

We now turn to the global maps *Π*_2_ and *Π*_4_ between neighbourhoods of the saddle points. For the heteroclinic connection from *p*_1_ to *p*_2_, we now consider the map $\Pi_{2}:\Sigma_{2}\to \Sigma_{3}$ which takes a point $\left({\hat{x}}_{1},d)\to ({\hat{x}}_{2},d\right)$ (where ${\hat{x}}_{2}<0$). For $0<{\hat{x}}_{1}\ll 1$ this takes the form

$${\hat{x}}_{2}=\alpha -A{\hat{x}}_{1}+O({\hat{x}}_{1}^{2}),$$

where the constant *A*>0 because the flow is two-dimensional, and the global bifurcation parameter *α* parametrizes the unfolding of the heteroclinic connection from *p*_1_ to *p*_2_. Similarly, to examine trajectories near to the heteroclinic connection from *p*_2_ to *p*_1_ we form the map $\Pi_{4}:\Sigma_{4}\to \Sigma_{1}$ which takes a point $\left(-d,{\hat{y}}_{2})\to (d,{\hat{y}}_{3}\right)$ such that

4.2 $${\hat{y}}_{3}=-\beta +B{\hat{y}}_{2}+O({\hat{y}}_{2}^{2}),$$

where *B*>0 is a constant and *β* is the bifurcation parameter. We now compose the maps to obtain $\Pi :=\Pi_{4}\circ \Pi_{3}\circ \Pi_{2}\circ \Pi_{1}:\Sigma_{1}\to \Sigma_{1}$ which takes the form

4.3 $$\begin{array}{r} {\hat{y}}_{3}=-\beta +Bd\;{\left(\frac{-\alpha +A{\hat{x}}_{1}}{d}\right)}^{c_{2}/e_{2}}, \end{array}$$

4.4 $$\begin{array}{r} =-\beta +\tilde{B}\;{\left[-\alpha +Ad\;{\left(\frac{{\hat{y}}_{0}}{d}\right)}^{c_{1}/e_{1}}\right]}^{c_{2}/e_{2}}, \end{array}$$

where $\tilde{B}$ is a rescaled constant. After rescaling to set as many inessential positive constants as possible to unity this is a one-dimensional map of the form

$$x_{n+1}=-\beta +a{\left(-\alpha +x_{n}^{\delta_{1}}\right)}^{\delta_{2}},\;\text{where}\;\delta_{i}:=\frac{c_{i}}{e_{i}}>0,\;\text{and}\;a>0,$$

and the new variable *x*_*n*_ is a new variable. Alternatively, we could write the map in two parts, in the form

4.5 $$y_{n+1}=-\alpha +{\hat{x}}_{n}^{\delta_{1}}\;\mathrm{and}\;x_{n+1}=-\beta +ay_{n+1}^{\delta_{2}}.$$

In the usual way, fixed point of this map indicate periodic, homoclinic and heteroclinic orbits for the ODEs. The conditions for the existence of various orbits (and their interpretation in the ODE dynamics) are therefore as follows:

(i) there exists a periodic orbit (a periodic wavetrain) if *y*=−*α*+*x*^*δ*_1_^>0 and *x*=−*β*+*ay*^*δ*_2_^>0;
(ii) there exists an orbit homoclinic to *p*_1_ (a slip pulse) if *x*=0 and *y*>0, i.e. 0=−*β*+*a*(−*α*)^*δ*_2_^ and *α*<0;
(iii) there exists an orbit homoclinic to *p*_2_ (a stick pulse) if *y*=0 and *x*>0, i.e. 0=−*α*+(−*β*)^*δ*_1_^ and *β*<0;
(iv) there exists a heteroclinic orbit from *p*_1_ to *p*_2_ (a detachment front) when *α*=0; and
(v) there exists a heteroclinic orbit from *p*_2_ to *p*_1_ (an attachment front) when *β*=0.

Figure 7*b* summarizes the curves and regions on which these orbit types exist; this figure then explains the qualitative nature of the ‘state diagram’ of slip and stick pulses and detachment and attachment fronts, near the codimension-two point P, as indicated in figure 6.

## 5. Discussion

We first provide a few comments on the necessity of our spinodal law, contrasting it with the use of monotonic or unregularized friction laws. We then make a few tentative physical remarks and suggest avenues for future work, before concluding by summarizing the results of the paper in a slighter wider context.

### (a) Results for simpler friction laws

A natural question to ask is whether all the ingredients of the spinodal friction law we chose are necessary to obtain the rich bifurcation structure of frictional waves we have found. For example, within rate-and-state friction theory, there is no *a priori* obvious choice for the state evolution function *G*, see [68,71]. The two most studied laws are known as the *Dieterich ageing law* and the *Ruina slip law* defined, respectively, by (with dimension)

5.1 $$G=G_{d}:=\frac{v\phi -L}{V_{*}}\;\text{and}\;G=G_{r}:=\left(\frac{v\phi }{V_{*}}\right)\;\ln\;\left(\frac{v\phi }{L}\right).$$

Regularized generalizations of the Dieterich–Ruina models such as the one introduced in §2 were proposed in [58] and shown to be consistent with many more physical phenomena.

We have repeated the analysis in this paper with simpler friction laws (results not shown). Note that for the Dieterich and Ruina laws, the shape of the friction laws *μ*_ss_(*v*) is monotonic so that only one equilibrium point of (3.13) is possible, corresponding to the creep uniform sliding state. Hence the planar slow–fast structure of (3.13) indicates that such a model allows only for wavetrains that have a form close to slip pulses. This solution type lies at the heart of many previous studies of the slip pattern formation along spatially homogeneous frictional interfaces (e.g. [31,82,92]).

Alternatively, if we introduce a spinodal version of the friction law without the hyperbolic regularization of the state evolution law, then we introduce both uniform stick and slip equilibria. However, both states are only able to support singular canard-type wavetrains leading to homoclinic orbits either of slip pulse or stick pulse type. Without the hyperbolic regularization of the state evolution law, the stiffness of the slow–fast dynamics is exacerbated and the wavetrain domain ii.WT_*m*_ is reduced to an exponentially thin region along the Hopf bifurcation locus H. The saddle–saddle connections of homoclinic and heteroclinic orbits are also confined along the H line.

We conclude that our regularized spinodal law, used here, is the most parsimonious model able to capture the range of dynamical phenomena we have uncovered in a single two-parameter state diagram.

### (b) Physical interpretation and future work

Before embarking on a discussion of physical interpretation, we should state at the outset that we have not yet considered the dynamics of the PDE system (3.4), nor established the stability of any of the wave structures we have constructed. Although the problem can be thought of as a generalized damped nonlinear wave equation, it has several non-trivial features, not least its slow–fast nature and that the state *u* is cyclic in the travelling-wave problem. We are unaware of any mathematical theory that can be applied directly to make generic statements about the dynamics and stability. We also need to consider boundary conditions carefully in order to describe a physical problem with either rigid or dead loading. Even straightforward time integration of the equations of motion requires careful treatment due to the numerical stiffness of the problem. Thus, any investigation into the dynamics and stability of the wave-train, pulse and front states is beyond the scope of the present study and will be left to future work.

Nevertheless, some preliminary remarks can be made. Firstly, the linear stability analysis of the uniform slip or stick states suggests that the Hopf bifurcations result in small amplitude waves that we expect to be stable on an infinite domain. Second, it would seem intuitive based on general theory (e.g. [93]), that one can indeed expect to find stable solutions to the nonlinear wave problem that are represented by connecting orbits between saddle points. However, such arguments are likely to be subtle owing to the multiple-time-scale nature of the nonlinearity in this model. An approach via geometric singular perturbation theory [84] may well prove feasible. We also point out that commonly used tools such as exponential dichotomies and Evans’ functions developed for reaction–diffusion systems (e.g. [93–97]) should be most useful to establish linear stability results. Finally, in close relation to our problem, we note that such results for wave-train solutions in the one-dimensional sine-Gordon and Klein–Gordon equations have recently been published [98,99].

**Figure 11.**
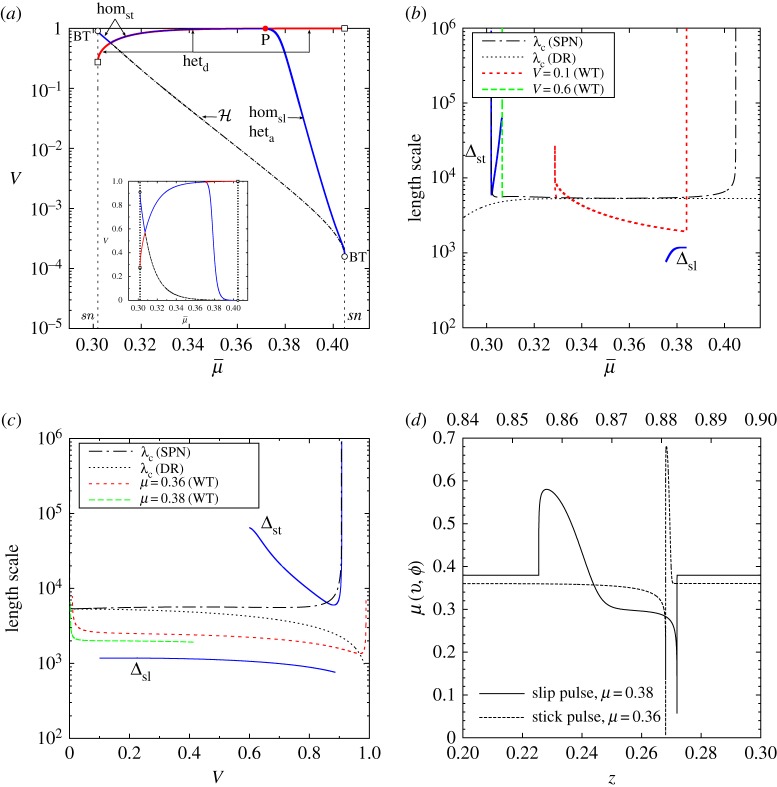
Replotting of figure 6 in the $\left(\bar{\mu },V\right)$ plane to show the characteristic wave speeds and length scales associated with the wave train, slip and stick pulse solutions. (*a*) The $\left(\bar{\mu },V\right)$ plane as in figure 6. (*b*) Length scales λ_*c*_, Δ as a function of $\bar{\mu }$: the blue solid lines correspond to hom_st_ (left) and hom_sl_ (right), and the dashed-dotted line indicates the variation in Δ along the Hopf bifurcation curve H. (*c*) Length scales λ_*c*_, Δ as a function of *V* . In (*b*) and (*c*), the red and green dashed lines indicate the characteristic length scale of the wave trains presented in figure 10. (*d*) Comparison of the frictional stress distribution along the slip and stick pulses.

Physical quantities, principally characteristic wave speeds and length scales, and hence time scales, can be extracted from our main results as presented in the $\left(V,\bar{\mu }\right)$ phase diagram in figures 6 and 7. To indicate these as clearly as possible, we focus on the curves of heteroclinic, homoclinic and Hopf bifurcations from figure 6 corresponding to the various localized and periodic structures. These curves also give the *stress–velocity relations* $V(\bar{\mu })$ of practical importance for each type of travelling wave (figure 11*a*).

Although existing over a range of values for *V* , it is useful to attempt to distinguish qualitatively between slow and fast propagating waves. An estimate of the order of magnitude of the wave speed can be obtained from the location of the BT points lying at the intersection of the Hopf (H) and saddle-node (*sn*) bifurcation curves. The location of these BT points is determined by the extrema of the friction curve and given by (4.1) and (2.5), where we set $V=\sqrt{r\zeta /(r\zeta +\gamma )}$ and use the appropriate values of *γ*, i.e.

5.2 $$\gamma (v_{\min})\approx \frac{ac^{2}}{{\left(b/a-1\right)}^{2}}\approx 2\times 10^{-7}\;\mathrm{and}\;\gamma (v_{\max})\approx bR^{2}{\left(1-\frac{a}{b}\right)}^{2}\approx 6\times 10^{3}.$$

Slip pulses and attachment fronts can be slow localized waves, whereas all types of localized waves can be fast. This does not contradict recent experimental and theoretical work reporting on the slow propagation of detachment fronts (e.g. [39,40,43,44,100]) as the order of magnitude of the wave speed *V* can be substantially reduced while increasing the confining pressure $\bar{\sigma }$, i.e. reducing *ζ*.

The characteristic length scales of periodic wave trains can be estimated from the neutral wavelength λ_*c*_ associated with the Hopf bifurcation. By contrast, we can compute numerical estimates of the length scale Δ associated with the two cases of slip and stick pulses by defining the integrals

$$\Delta_{\mathrm{sl}}:\;=\;\left(\frac{V}{r}\right)\int_{0}^{T}\frac{\left[v(z)-\min(v)\right]}{\delta v}\;dz\;\mathrm{and}\;\Delta_{\mathrm{st}}:\;=\;\left(\frac{V}{r}\right)\int_{0}^{T}\frac{\left[\max(v)-v(z)\right]}{\delta v}\;dz,$$

where the first definition holds for slip pulses and the second for stick pulses, and we denote by *δv* the interfacial velocity jump, defined as δv:=max(v)−min(v). Figure 11*b*–*c* illustrates these length scale estimates based on the stress-velocity curves in figure 11*a*. We see that characteristic sizes of the slip and stick pulses deviate from the neutral wave train wavelength λ_*c*_ by a minimum of one order of magnitude. Note that the lines for Δ_*st*_ and Δ_sl_ end where the computations break down due to the numerical stiffness of the problem in AUTO [85,86]. Further, we note that the orders of magnitude of these length scales are compatible with the long-wavelength assumption underlying this work.

Finally, we comment on the friction stress distribution along the interface for the slip and stick pulses as shown in figure 11*d*. For the slip pulse case, the spatial variations of *μ* are localized within the pulse itself, which contrasts with crack-like models characterized by a stress singularity at the front of the pulse [29,30]. Our friction law truly generates a self-healing pulse as the friction stress in front of, and behind, the pulse zone is just the applied shear stress $\bar{\mu }$, the slip rate being equal to that of the corresponding ‘stick’ equilibrium. This is a consequence of the spinodal nature of the friction model we use. For the stick pulse, the friction stress distribution mimics a crack solution with a stress singularity at the front of the ‘stick’ zone, which is associated with a diffuse drop in the slip rate.

It is worth pointing out that the stress–velocity relation of the detachment fronts, i.e. het_d_, is qualitatively equivalent to the $V(\bar{\mu })$ curve computed in [44] (figure 4). In their somewhat different non-monotonic friction model, its local minimum sets a lower bound, equivalent to our *V*_min_, for a rupture front propagation speed, which is consistent with experimental measurements in [40]. Our analysis also attributes a clear definition of *V*_min_ as the front speed of the codimension-two non-central saddle-node heteroclinic bifurcation point (open squares). Similarly, the local maximum of our friction law, gives a maximum *V*_max_<1 for the front speed which is below the slab speed of sound.

Further physical consequences of these results for earthquakes mechanics and engineering systems remain to be explored and are left for future work. However, this work reveals that the quantitative features of the stress-velocity curves strongly depend on the mathematical details of the state evolution law, whose experimental identification and theoretical derivation from first principles are still open questions.

### (c) Summary

A comprehensive understanding of the physical mechanisms that determine the diversity of frictional slip pattern formation along extended contacts between solids that has been reported over the years [40,42,82] is still lacking. This lack would appear to be at least in part because of incomplete physical and mathematical modelling of friction and how it couples with elastic wave radiation. A key phenomenon we have sought to explain is that such interfaces may possess, depending on the stress scales involved, either waves of slip on a background of uniform stick or creep, or waves of stick within a background of uniform slip. We have also explained that such waves can exist as isolated pulses, as periodic waves or as fronts between regions of homogeneous slip or stick.

Specifically, this work has shown how introducing a smooth interfacial friction model can explain the origin of slip pulses, stick pulses, travelling wavetrains, detachment fronts and reattachment fronts, all in the same mathematical formulation of regional contact and within the well-established theory of smooth dynamical systems theory. The key ingredient of the mathematical model is that the friction law should present non-monotonic velocity-dependent steady-state characteristics. Then, by assuming deformation in the form of a steady travelling wave, it is argued that slip and stick pulses can be understood in terms of homoclinic global bifurcations of travelling periodic slip patterns, as well as the travelling fronts as heteroclinic connections.

We emphasize that slip pulses are anchored at the equilibrium saddle point lying on the low velocity strengthening branch of the steady-state friction curve, while the existence of a high velocity strengthening branch in spinodal friction also allows the existence of ‘stick pulse’ which corresponds to a narrow travelling ‘stick’ zone. Along the bifurcated branch, travelling wave trains of slip pulses develop from a canard explosion, which can lead to relaxation oscillations. We note that the qualitative features of these patterns are independent of their propagation direction with respect to the slab motion. More broadly, we have shown how this plethora of behaviours is shown to be a consequence of the spinodal character of friction, by showing that simpler friction models are unable to reproduce this behaviour.

Finally, we stress that the localized pulse solutions exist only along specific lines in parameter space, giving stress–velocity relations and separating domains of generic travelling fronts and wavetrains of various types. This may contribute to a better understanding of why friction experiments are so notoriously difficult to perform in an experimentally reliable and repeatable way (e.g. [9]). For instance, our analysis may provide a possible origin for the scattering in experimental measurements of the detachment fronts’ speed [40], the experimental apparatus not only sampling proper heteroclinic detachment fronts but also the generic detachment fronts belonging to the adjacent regions of the heteroclinic bifurcation curve. In this respect, in any specific experiment, initial and boundary conditions also will play a key role in the selection of solutions; we will return to this rich avenue for investigation in future work.

## Data accessibility

All data are contained within the published paper.
